# The Effect of Sodium Dodecyl Sulfate (SDS) and Cetyltrimethylammonium Bromide (CTAB) on the Properties of ZnO Synthesized by Hydrothermal Method

**DOI:** 10.3390/ijms131013275

**Published:** 2012-10-16

**Authors:** Donya Ramimoghadam, Mohd Zobir Bin Hussein, Yun Hin Taufiq-Yap

**Affiliations:** 1 Advanced Material and Nanotechnology Laboratory (AMNL), Institute of Advanced Technology (ITMA), Universiti Putra Malaysia, 43400 UPM, Serdang, Selangor, Malaysia; E-Mail: dona_textile1984@yahoo.com; 2 Department of Chemistry, Faculty of Science, Universiti Putra Malaysia, 43400 UPM, Serdang, Selangor, Malaysia; E-Mail: yap@science.upm.edu.my

**Keywords:** zinc oxide, sodium dodecyl sulfate, cetyltrimethylammonium bromide, hydrothermal synthesis

## Abstract

ZnO nanostructures were synthesized by hydrothermal method using different molar ratios of cetyltrimethylammonium bromide (CTAB) and Sodium dodecyl sulfate (SDS) as structure directing agents. The effect of surfactants on the morphology of the ZnO crystals was investigated by field emission scanning electron microscopy (FESEM) and transmission electron microscopy (TEM) techniques. The results indicate that the mixture of cationic-anionic surfactants can significantly modify the shape and size of ZnO particles. Various structures such as flakes, sheets, rods, spheres, flowers and triangular-like particles sized from micro to nano were obtained. In order to examine the possible changes in other properties of ZnO, characterizations like powder X-ray diffraction (PXRD), thermogravimetric and differential thermogravimetric analysis (TGA-DTG), FTIR, surface area and porosity and UV-visible spectroscopy analysis were also studied and discussed.

## 1. Introduction

In past decades, ZnO nanostructures have attracted a considerable attention due to their unique properties and various applications such as photo catalysts, conductivity, gas sensor, chemical sensors *etc.* [1–4]. ZnO is also a useful semiconductor material due to the wide direct band gap (3.3 eV) and large value of excitation binding energy (60 meV).

Synthesis of ZnO nanostructures is subject to intense research lately due to very rich shapes such as wires, tubes, rods, flowers, sheets/flakes, particles, stars, belts and hexagonal prismatic using various methods such as thermal evaporation, chemical vapor deposition and wet chemical process like template-based, surfactant-assisted precipitation, hydrothermal and solvothermal methods [5–10].

Among these methods, hydrothermal is one of the most common and promising methods for the synthesis of isometric zinc oxide crystals. This method enjoys advantages such as a one-step synthesis without any additional process like calcinations and milling; low level of aggregation, high purity, nonpolluting, inexpensive and narrow particle size distribution. Moreover the hydrothermal method, due to the low-temperature reaction in water under a sealed environment is consistent with the green chemistry. The only drawback is that the products prepared in aqueous solution are usually poor in terms of shape and size control. Fortunately, surfactants coupled with hydrothermal method are able to modify the surface chemistry of nanomaterials by changing their hydrophobic or hydrophilic properties. Therefore, the desired size and shape can be tailor-made.

Recently, there have been lots of works on the synthesis of ZnO nanostructures using different surfactants like ethylenediaminetetraacetic acid (EDTA) [11], sodium dodecyl sulfate (SDS) [12], cetyltrimethylammonium bromide (CTAB) [13], polyethylene glycol (PEG) [14] and polyethyleneimine (PEI) [15,16]. These studies mostly investigated the effect of surfactants on the morphology of ZnO crystals separately. Different shapes like nano-wires, flowers, rods, hexagonal bipyramid and microspheres have been achieved by SDS or CTAB individually.

There are few studies in which mixture of CTAB and SDS has been applied [17–20]. ZnO nanostructures with the morphology of crooked anomalous nanosheets and very unique aggregated nanosheets have been obtained in the presence of SDS/CTAB surfactants via a mild hydrothermal process and precipitate method, respectively. However, there is still a lack of adequate information about ZnO properties.

Therefore, the effects of CTAB and SDS mixture as surfactants on the surface morphology surface and other properties of ZnO nanostructures will be investigated. The hydrothermal method was adopted in the synthesis.

## 2. Results and Discussion

### 2.1. XRD Analysis

**Figure 1:**
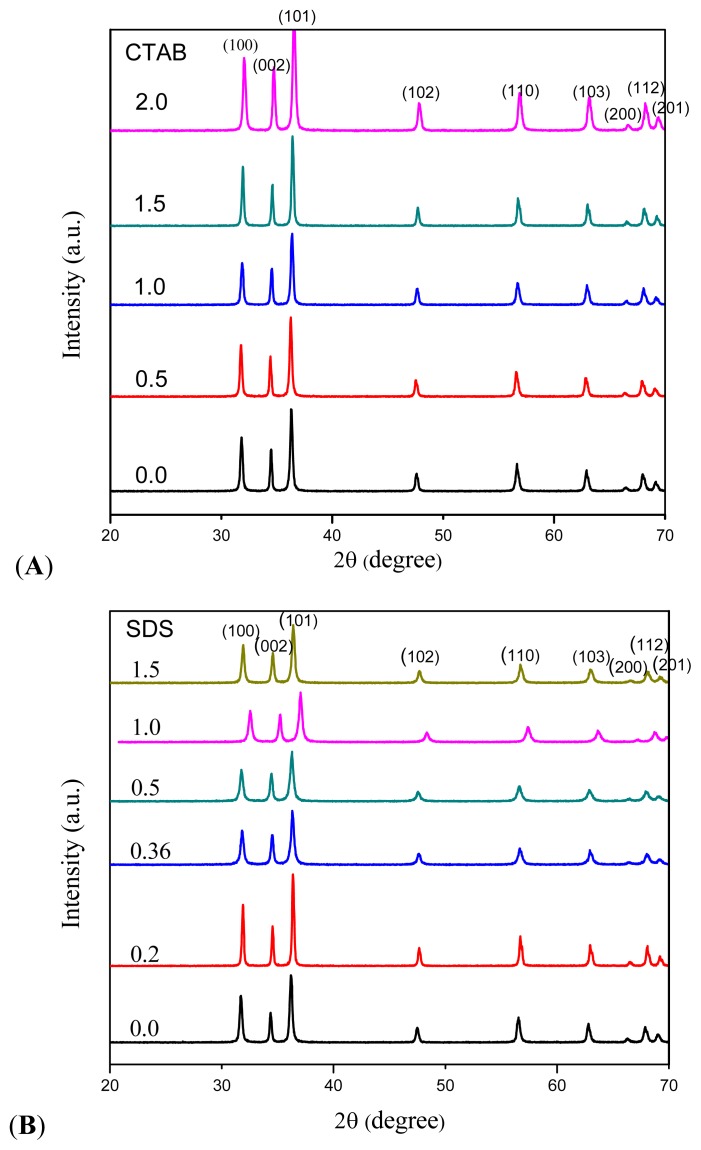
Powder X-ray diffraction (PXRD) patterns of as-prepared ZnO synthesized at different mole ratios of (**A**) cetyltrimethylammonium bromide (CTAB) (0, 0.5, 1, 1.5, 2) and (**B**) sodium dodecyl sulfate (SDS) (0, 0.2, 0.36, 0.5, 1, 1.5).

The XRD patterns of the as-synthesized products using CTAB and SDS by hydrothermal method are shown in Figure 1. All diffraction peaks can be indexed as hexagonal wurtzite-structure (JCPDS card No. 36-1451). The sharp and narrow peaks also illustrate that ZnO particles enjoy high crystallinity and purity. Samples synthesized with constant amount of SDS followed by addition of different molar ratios of CTAB show higher intensity compared to the sample prepared with the present of SDS only. Similar results was observed by Tan *et al.* [18], in which reflections of the lamellar phase constructed by mixture of SDS and CTAB show significantly increase in intensities (three times) compared to those produced by the present of SDS only. In fact, CTAB interacts with the SDS-bilayer assemblies and affects their packing and degree of ordering. This is due to different degrees of preferred growth orientation along the c-axis of the ZnO phase [21–23]. In addition, in cases of samples prepared at SDS:CTAB = 1:2 and CTAB:SDS = 1:0.2, in which CTAB concentration is higher than SDS, higher intensity of X-ray reflections was observed, indicating high crystallinity of the resulting samples.

### 2.2. Morphology

**Figure 2:**
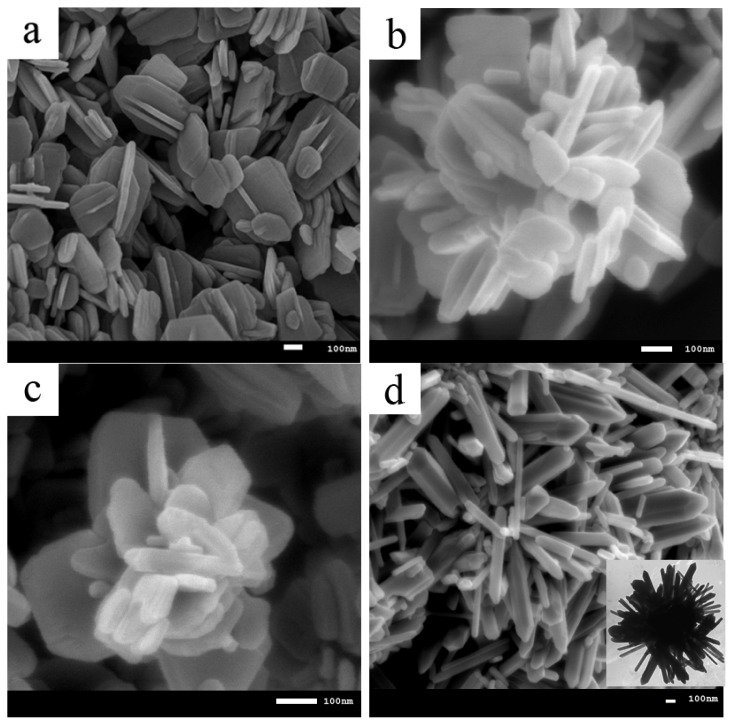

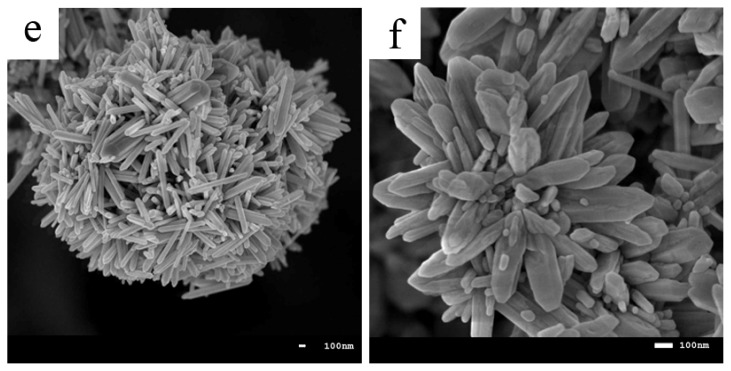
Field emission scanning electron microscopy (FESEM) images of ZnO prepared using different mole ratios of CTAB to SDS, (**a**) SDS:CTAB = 1:0; (**b**) SDS:CTAB = 1:0.5; (**c**) SDS:CTAB = 1:1; (**d**) SDS:CTAB = 1:1.5; (**e**,**f**) SDS:CTAB = 1:2, 2d (inset) transmission electron microscopy (TEM) image of SDS:CTAB = 1:1.

Figure 2a–f show field emission scanning electron microscopy (FESEM) images of as-synthesized ZnO nanoparticles at different molar ratios of CTAB to SDS with constant amount of SDS.

To investigate the effects of cationic/anionic surfactants on ZnO morphology, each surfactant was used individually. ZnO-SDS particles shown in Figure 2a are mostly nanoflakes and containing few nanorods similar to previous study [21]. Samaele *et al.* [10] obtained ZnO-SDS nanorods agglomorated with sphere-like particles by aqueos solution. Sun *et al.* [12] gained ZnO-SDS nanowires with high aspect ratio by microemulsion method. The as-synthesised ZnO-SDS nanoparticles calcined at 500 °C shows no significant difference compared with that of the uncalcined one.

Adding CTAB to the mentioned solution has noticeably affected the morphology of particles. Aggregated nanosheets can be observed with the existence of CTAB with molar ratio of 0.5 as seen in Figure 2b. Usui [19] have reported almost same morphology for ZnO crystals grown with the mixture of CTAB/SDS by precipitation method. Increasing the CTAB molar ratio from 0.5 to 1 and 1.5 alter the morphology to rose-like structure and hexagonal-shaped rods form spherical outline as shown in Figure 2c,d, respectively. Detailed view on the rose-like structure indicates that it is comprised of many nanoflakes and nanoparticles. The hexagonal-shape rods form spherical outline ZnO is clear in the inset of Figure 2d, which shows TEM image. When the molar ratio of SDS:CTAB = 1:2, some sphere outline consisting rods and flower-like (hyacinth-like) structures coexist in the product (see Figure 2e,f).

Samples prepared with no CTAB and low concentration of CTAB, the length of flakes and sheets were in the range of 80–500 nm while for sample prepared using SDS:CTAB = 1:1.5 rods with length of 2–3 μm and diameter of 50–250 nm were observed. With increasing the CTAB molar ratio to 2, length and diameter of the rods both decreased to 1 μm and 50–150 nm, respectively. The decrease in diameter of nanorods can be described as follow. The negatively charged SDS ions assembled and form spherical micelles, which encapsulate ZnO seeds. This encapsulation restricts the access of zinc ions to the ZnO seeds. This process leads to formation of nucleation sites of new ZnO rods growing randomly at the outer edge of micelles. When CTAB was added, the surfactant may cap the ZnO nanorods to inhibit further lateral growth. Therefore, the increased of CTAB concentration limits the lateral growth of ZnO rods and decreased the diameter and length of ZnO crystals. Similar results on lateral growth restriction of ZnO nanorods were previously reported by Maiti *et al.* [22].

**Figure 3:**
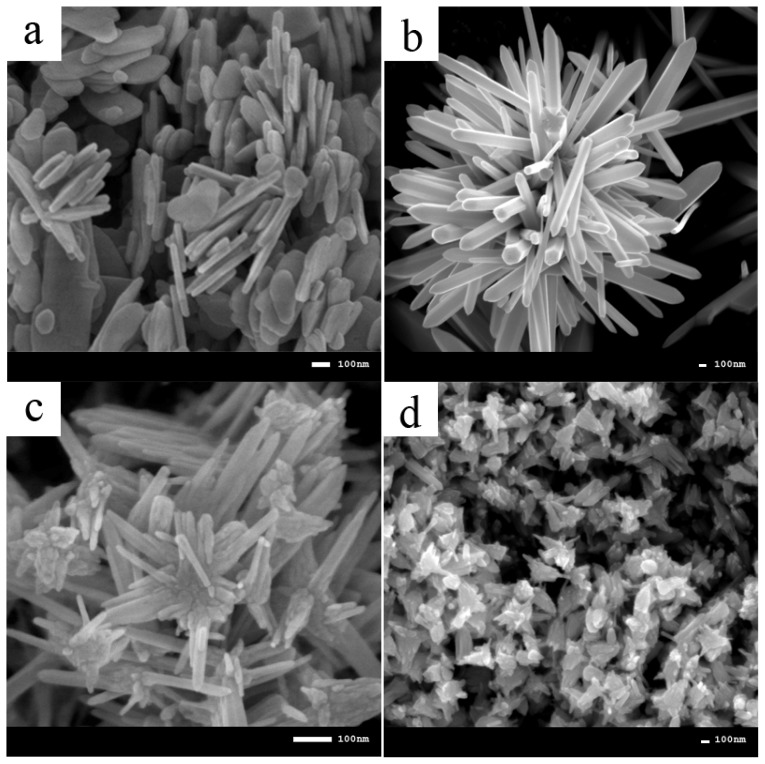

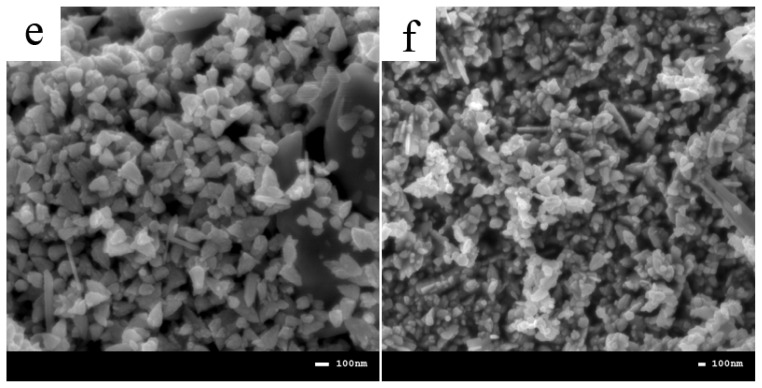
SEM images of ZnO crystals prepared at (**a**) CTAB:SDS = 1:0; (**b**) CTAB:SDS = 1:0.2; (**c**) CTAB:SDS = 1:0.36; (**d**) CTAB:SDS=1:0.5; (**e**) CTAB:SDS = 1:1 and (**f**) CTAB:SDS = 1:1.5.

Figure 3a–f show FESEM images of as-obtained ZnO nanostructures at different molar ratios of SDS to CTAB.

It was found that nanoflaked-shape obtained in presence of CTAB, while by adding little amount of SDS, hexagonal-shaped rods form spherical outline were observed. The length of nanorods are in the range of 100 nm to 2 μm and diameter varies, but below 200 nm. With increasing of SDS molar ratio from 0.2 to 0.36, another morpholgy has revealed which is branch rod-like structure. The length and diameter decreased dramatically to the range of 100–300 nm and 15–50 nm, respectively. Most of rods are agglomorated and seems thicker. The nano- and micro-sized structures can be observed in Figure 3c.

Figure 3d–f show ZnO synthesized at different molar ratios of SDS; 0.5, 1 and 1.5, respectively. The morphology is based on the branch rod-like nanostructures but more agglomoration can be easily observed. Rod’s shape is not clear and nanoparticles look like a triangular structure.

The origin of different morphology can be elaborated by a chemical interaction between the ZnO seed surface and ions produced by the ionization of the SDS and CTAB [19]. The growth mechanism of ZnO crystal is suggested as follows.

The results showed that by adjusting the ratio of the surfactants, SDS to CTAB, we are able to control the physico-chemical properties of the resulting samples. In the case of constant amount of SDS followed by addition of CTAB, the interactions between the zinc species, ([Zn(OH)_4_]^2−^) and SDS in the alkaline media are mediated by CTAB cationic surfactant, in which an increase in cationic concentration can significantly promote the interactions between zinc species and SDS [23]. As it can be seen from Figure 2a–f, the morphology of ZnO nanostructures changed from disordered flake-shapes to rod-, flower- and sphere-like structures.

In the case of the synthesis using a constant amount of CTAB, the addition of SDS as a second surfactant would control the electrostatic interaction between zinc species ([Zn(OH)_4_]^2−^) and cationic part of CTAB on the surface of formed micelles. SDS due to its sulfate ion can change the geometry of CTAB micelles and suppresses the growth of ZnO crystals [24]. Thus ZnO nanoparticles with smaller size are obtained. As seen in Figure 3a–f, the increase of the SDS amounts in the mother liquor, resulted in different environments of interaction between CTA^+^ and zinc species, and subsequently resulted in different morphologies of the ZnO, from rods-spherical outline and branched rod-like to nanoparticles shapes.

**Figure 4:**
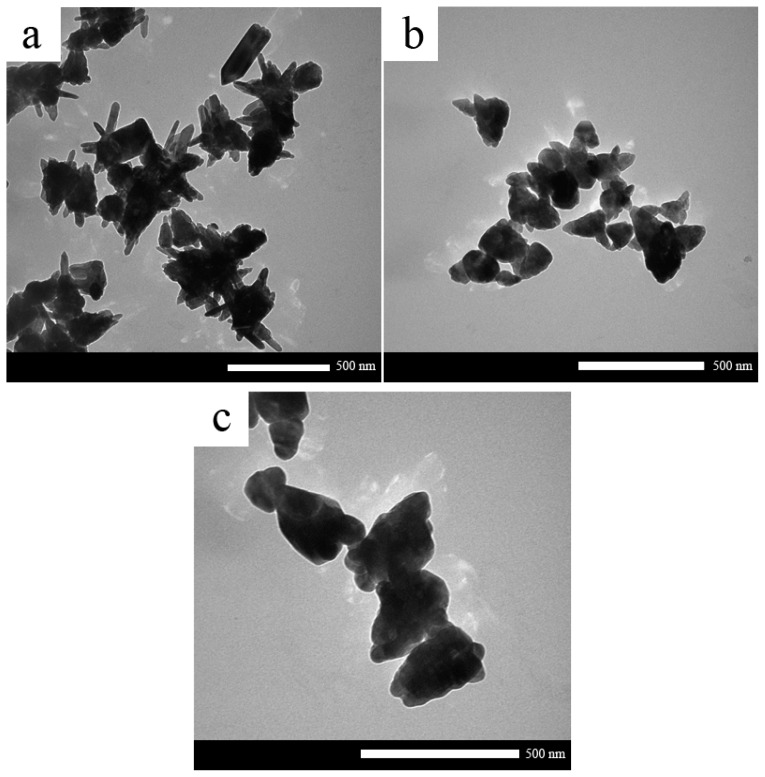
TEM images of as-synthesized ZnO nanostructures with (**a**) CTAB:SDS = 1:0.5, (**b**) CTAB:SDS = 1:1 and (**c**) CTAB:SDS = 1:1.5.

Figure 4 shows TEM images of ZnO nanostructure synthesized at molar ratios of CTAB:SDS = 1:0.5, 1:1 and 1:1.5. The average size of particles decreased from 300 nm to 150 nm and then 100 nm with increasing the amount of SDS. Therefore at specific molar ratios of SDS to CTAB, ZnO nanoparticles were observed.

### 2.3. Thermal Analysis

**Figure 5:**
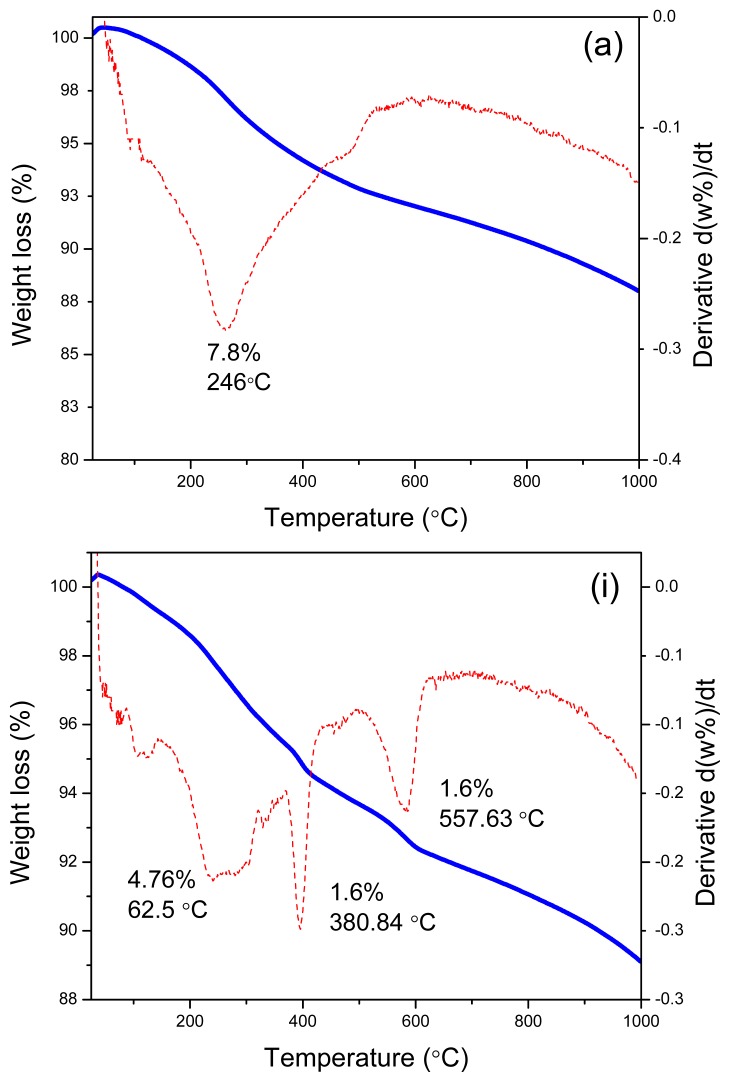

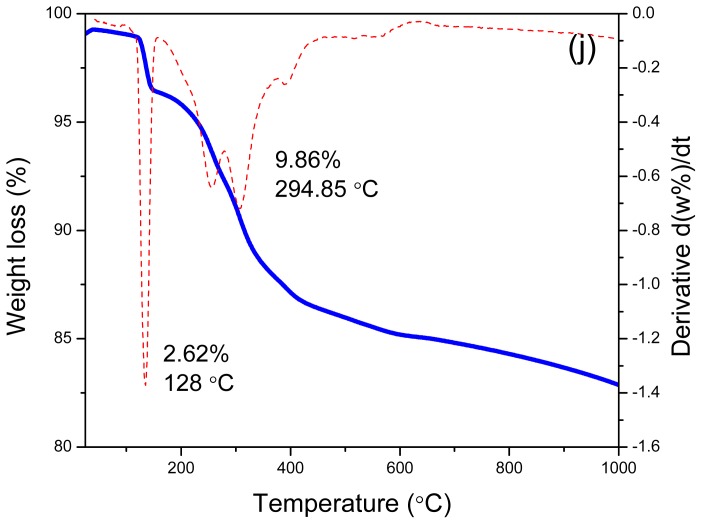
Thermogravimetric and differential thermogravimetric analysis (TGA-DTG) thermogravimetric analysis of as-synthesized ZnO samples prepared at different mole ratios of (**a**) SDS:CTAB = 1:0; (**i**) CTAB:SDS = 1:0.5; (**j**) CTAB:SDS = 1:1.

Thermal analysis of the as-synthesized products is shown in Figure 5 for samples synthesized at different molar ratios of CTAB and SDS. (The rest of Thermogravimetric and differential thermogravimetric analysis (TGA-DTG) plots can be found in the supplementary information).

Most of the TGA/DTG curves present similar behavior with one main weight loss step in the temperature range of 30–500 °C including the dehydration of physically adsorbed water from 30–200 °C and decomposition of surfactants from 200 to 500 °C [25]. Thermal degradation of pure SDS and CTAB were seperately recorded, showing a loss of between 160–380 °C and 180–340 °C, respectively. However it can be easily interpreted from the curves that the remaining weight did not level off even near to 1000 °C. Therefore there is possibility of gradually decomposition of surfactants and shifting to higher temperature. As seen for sample CTAB:SDS = 1:0.36 in Table 1, the offset temperature of surfactants decomposition has shifted to 934 °C.

As shown in the Table 1, two weight loss steps were obeserved in ZnO samples prepared at SDS:CTAB = 1:0.5 and 1:2. The temperature range of decomposition for sample prepared at molar ratio of 0.5 is about 60–500 °C which is very close to the rest of samples. For the sample with the molar ratio of 2, the first weight loss of 1.7% is described as removal of bonded water and the second weight loss of 5.3% observed between 424 and 948 °C due to the surfactants removal. Samaele *et al.* [10] reported almost similar temperature (577–900 °C) for SDS-modified ZnO particles via the precipitation method. The higher temperature for surfactant degradation can be attributed to relatively high heating rate and the quality of the material. Denoyel *et al.* [26] claimed that reducing the heating rate from 10 to 1 °C/min can definitely shift the TGA curves to lower temperatures.

The only three steps of weight loss was assigned for sample prepared at CTAB:SDS = 1:0.5 with temperature range of 30–349 °C, 355–464 °C and 464–599 °C which are corresponding to two weight losses as discussed above (30–500 °C). On the basis of a little temperature differences, there is another possibility of partial degradation of surfactant, meaning that in the first step, the surfactant started to degrade right after dehydration of water but incomplete, and again in second step the residual surfactants decomposed and so on until entire degradation taken place [26].

### 2.4. FTIR Spectroscopy

**Figure 6:**
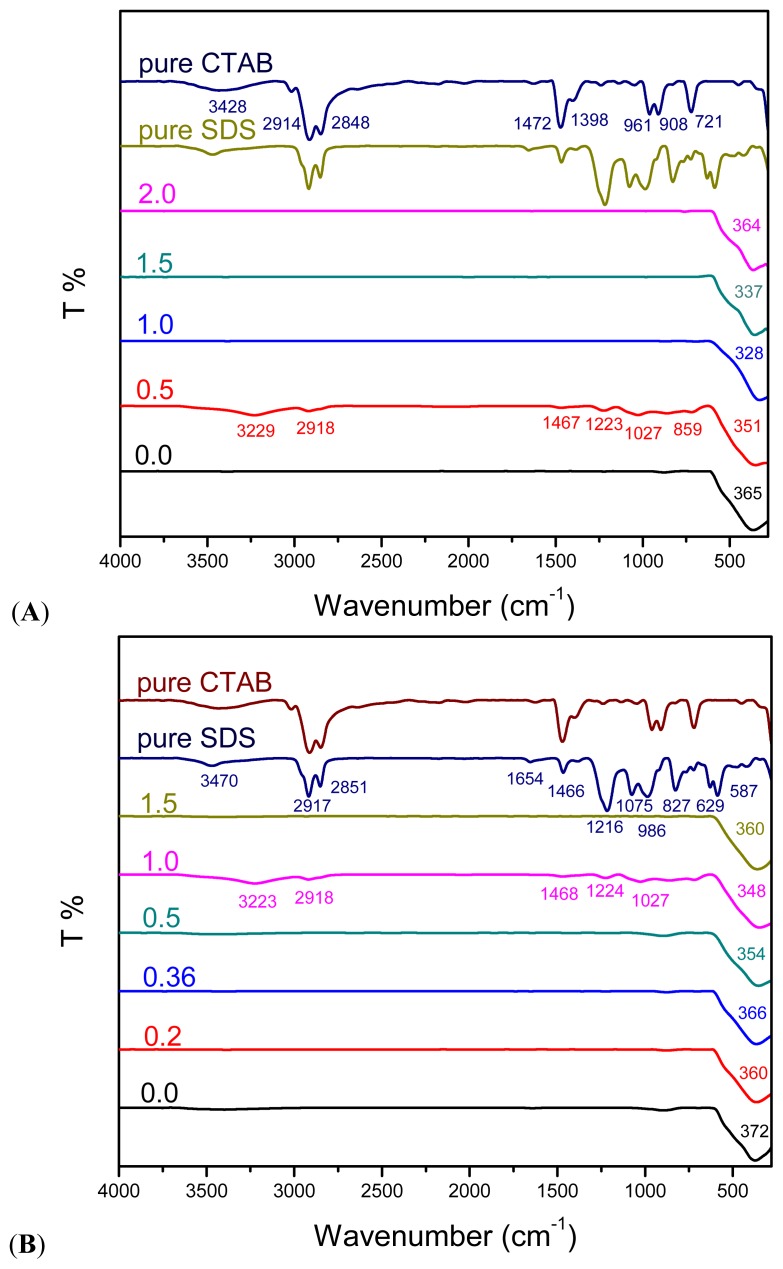
FTIR spectra of as-produced ZnO samples with constant amount of SDS and different mole ratios of (**A**) CTAB (SDS:CTAB); (**B**) SDS (CTAB:SDS).

**Figure 7:**
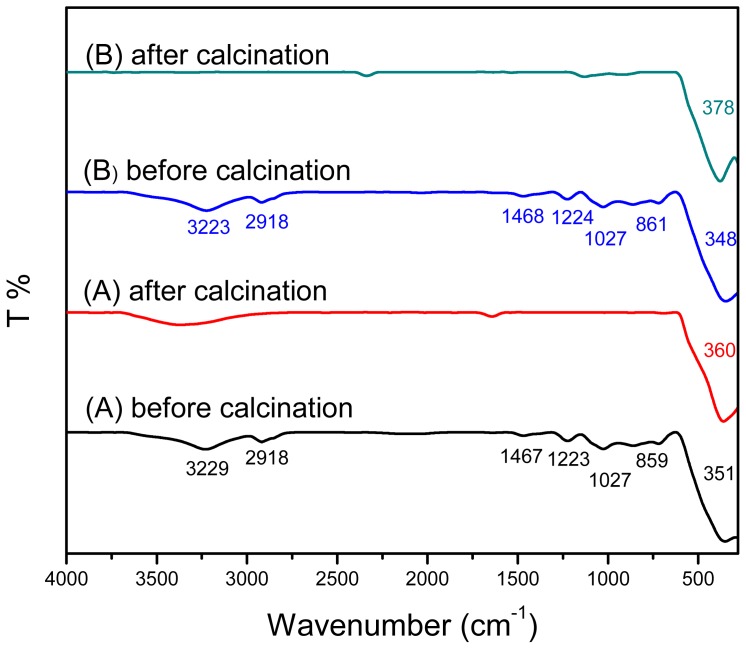
FTIR spectra of as-produced ZnO samples at different mole ratios of (**A**) SDS:CTAB = 1:0.5 and (**B**) CTAB:SDS = 1:1 before and after the calcinations at 500 °C for 5 h.

To study the adsorption of surfactants on the surface of ZnO nanostructures, FTIR spectrum was recorded in the range of 4000–280 cm^−1^ (Figure 6A,B). It is notable that most of FTIR spectra show no characteristic peaks of CTAB or SDS which is in a good agreement with the XRD results previously discussed. Samples prepared using SDS:CTAB = 1:0.5 (Figure 6A) and CTAB:SDS = 1:1 (Figure 6B) are exceptional which demonstrate quite similar bands at 3229, 2918, 1468, 1224, 1027 and 860 cm^−1^ corresponds to water O–H stretching vibration, C–H stretching and bending, S=O stretching vibration of SO_4_ from SDS molecule and C–H stretching, respectively [27–29]. Indeed these peaks are assigned to chemical groups at the particle surface [27], which cannot be easily removed during the washing process, but can be easily removed through calcinations. As seen in Figure. 7, the characteristic peaks of surfactants obviously disappeared after calcinations at 500 °C for 5 h.

The results from TGA show total weight loss from 2% to 12.5% including decomposition of adsorbed surfactants. Therefore, the absence of the adsorption of CTAB and SDS molecules in the corresponding FTIR spectra may be due to the trace content of CTAB and SDS molecules adsorbed on ZnO structures.

The characteristic peak of ZnO absorption can be clearly observed at 330–370 cm^−1^ which is less than that of reported in literatures. Wu *et al.* [30] reported two maximum characteristic absorption peaks for ZnO nanorods using CTAB as surfactant at 512 cm^−1^ (blue-shifted) and another with wavenumber shorter than 500 cm^−1^. Samaele *et al.* [10] and Prasad *et al.* [27] also reported absorption peak of 470 cm^−1^ and 422 cm^−1^ for SDS-modified ZnO particles and nanorods, respectively. It seems that mixture of these cationic-anionic surfactants shifted the characteristic peak of ZnO to lower wavenumbers (blue-shifted) as seen in Figure 6A,B. It is worth to mention that calcination treatment could shift characteristic peaks of ZnO back to higher wavenumbers (Figure 7).

## 2.5. Surface Properties

**Figure 8:**
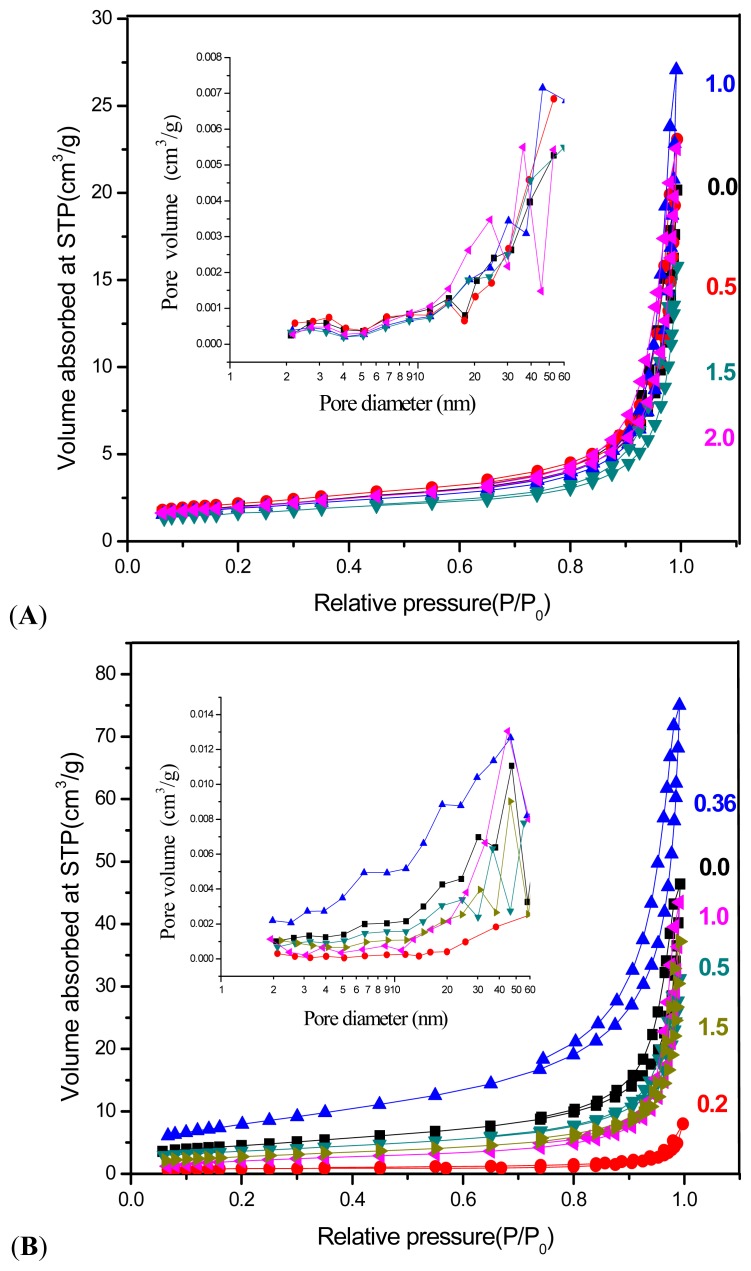
Nitrogen adsorption-desorption isotherms and Barret-Joyner-Halenda (BJH) pore size distribution of as-obtained ZnO nanostructures with different mole ratio of (**A**) CTAB and (**B**) SDS.

Figure 8 shows adsorption-desorption isotherms and BJH pore size distribution for ZnO nanostructures prepared at different molar ratios of surfactants.

Both isotherms, Figure 8A,B can be ascribed as Type IV, which is associated to capillary condensation according to IUPAC classification with Type H3 hysteresis that is characteristics of the mesoporous material with slit-shape pores. On the basis of results from Figure 8A, the absorption of all ZnO products gradually increased from low pressure of about 0.06 to 0.8, and then followed by a sharp rise from 0.8 and above due to substantial interparticle porosity [31]. The most and the least volume absorbed are assigned to samples with molar ratio of 1:1 and 1:1.5, respectively. The desorption branch are quite similar corresponding to similarity in their pore’s texture [32]. A detailed view on the Figure 8A,B show that all the isotherms in the Figure 8B indicated greater increase at low pressure around 0.06 to 0.65 followed by steep slope from 0.65 above in the volume adsorbed compared to Figure 8A. Among all the samples, sample prepared at CTAB:SDS = 1:0.36 showed the highest volume adsorbed and widest desorption branch as seen in the Figure 8B.

Barret-Joyner-Halenda (BJH) pore size distribution for as-synthesized ZnO nanostructures is shown in Figure 8A,B (inset). Both plots are located in the mesopore’s range, which is in agreement with the adsorption isotherm of Type IV. BJH pore size distribution of ZnO with SDS and CTAB (Figure 8A inset) indicates different features to that of ZnO synthesized with no CTAB (containing SDS only), specifically from pore diameter around 18 nm above. This is due to the result of modification in pore texture. Similar result can be identified from Figure 8B (inset) in which pore size distribution of CTAB:SDS = 1:0.36 and 1:0.2 proves pore texture modification in comparison with product included CTAB only. On the basis of these observations we can suggest that mixture of CTAB and SDS with certain molar ratio of each can modify the pore characteristics. This result was found consistent with results from Table 2.

The BET surface area and the average pore diameter and pore volume of ZnO nanostructures are listed in Table 2. As seen from the table, the average pore diameter for the as-obtained products is between 16 to 23 nm. Hu *et al.* [33] reported that pores around 19 nm are assigned to the interparticle space, arising from the assembly of the large individual porous ZnO particles, which is in good agreement with results from adsorption isotherm mentioned earlier. From surface area values, the consequence can be derived that increasing CTAB in ZnO samples with constant amount of SDS could not considerably change the surface property of products while adding SDS to ZnO with a constant amount of CTAB enhanced the surface area up to 29 m^2^/g. In fact, surface area is inversely related to particle size. In other words, specific surface area increase with the decrease of particle size as well as pores presence, which lead to the increase in the specific surface area. As seen in Table 2, in some cases a decrease in surface area can be observed although particle size decreased. There are other possibilities that may affect the surface area value. In the case of CTAB molar ratio at 2 and 0.5, the decrease in surface area might be due to a shift in the particle size distribution to a larger mean size [34]. As discussed in the related section, size of particles increased with the increased of the CTAB. In the case of SDS molar ratio at 0.36 and 1.5, the decrease of surface area may take place due to the collapse of pore structure [35]. In fact, collapse of the interconnected mesopores enlarged and changed the smaller mesopores into irregularly shaped larger mesopores and decreased the surface area value, as expected.

The effect of reaction temperature on the surface area of two ZnO samples was also investigated and results are listed in Table 3. Table contents show that increasing the reaction temperature decreased the surface area of the as-obtained ZnO nanostructures and the values for samples prepared in both temperatures, 150 °C, 180 °C are almost similar.

## 3. Experimental Procedures

### 3.1. Reagent

All chemicals used in this work were of analytical reagent grade and used as received without any further purification. All the aqueous solutions were prepared using deionized water.

### 3.2. Hydrothermal Growth

#### 3.2.1. Synthesis of ZnO Using CTAB (Constant) and SDS (Variant)

In a typical procedure, 1 g of zinc acetate (Zn(Ac)_2_ 2H_2_O) and 2g cetyltrimethylammonium bromide (CTAB, C_19_H_42_BrN) were dissolved in 60 mL distilled water under constant stirring. Second, 5 mol/L NaOH solution was gradually introduced to the mentioned solution until pH reached at 13. Then, certain amount of sodium dodecyl sulfate (SDS, C_12_H_25_SO_4_Na) was added to the solution as molar ratio of CATB: SDS equaled to 1:0, 1:0.2, 1:0.36, 1:0.5, 1:1 and 1:1.5. Finally, solution with white flocculent precipitate was transferred into a teflon-lined stainless steel autoclave 50 mL and hydrothermal growth was carried out at 120 °C for 18 h. After treatment, the autoclaves were allowed to cool down and the white precipitates were collected, centrifuged at 40,000 (× g) for 10 min and supernatant was discarded. The obtained particles were washed three times with ethanol and distilled water, in order to remove impurities and dried at 60 °C for 24 h.

#### 3.2.2. Synthesis of ZnO Using SDS (Constant) and CTAB (Variant)

Firstly 1 g zinc acetate (Zn(Ac)_2_ 2H_2_O) and 1.12 g NaOH were added in 25 mL distilled water and the solution was stirred. After 1 h, 0.1 g SDS was added to the solution. Then, certain amount of CTAB was added to above solution so that the molar ratio of SDS:CTAB was set to 1:0, 1:0.5, 1:1, 1:1.5 and 1:2. The rest of the process is the same as was mentioned earlier in the synthesis of ZnO using CTAB and SDS (Section 2.2.1).

### 3.3. Characterization

Powder X-ray diffraction (PXRD) analysis was performed on a Shimadzu diffractometer, XRD-6000 (Tokyo, Japan) equipped with CuKα radiation. The morphology of the micro and nanostructures were characterized by field emission scanning electron microscopy (FESEM) JOEL JSM-6400 (Tokyo, Japan) and transmission electron microscopy (TEM) Hitachi H7100. Surface characterization of the material was carried out using nitrogen gas adsorption–desorption technique at 77 K using a Micromeritics ASAP 2000 (Norcross, GA, USA). Thermogravimetric and differential thermogravimetric analysis (TGA-DTG) were carried out using a Mettler Toledo instrument (Greifensee, Switzerland) using heating rate of 10 °C/min, in the range of 25–1000 °C under a nitrogen atmosphere. Fourier transform infrared spectra were recorded over the 280–4000 cm^−1^ range using a Perkin-Elmer 100 spectrophotometer (Waltham, MA, USA) under standard conditions. Ultraviolet-visible spectra were used to determine the optical properties.

## 4. Conclusions

The mixture of CTAB and SDS surfactants changed the morphology of ZnO crystals from mostly nanoflakes to well-defined structures such as rods, spheres, flower-like and triangular-like nanoparticles. However, a more considerable effect was observed in samples synthesized at a constant concentration of CTAB and various concentrations of SDS, due to overall conversion of structure from nanoflakes to nanoparticles. It is noteworthy that the weight loss of approximately 10% was determined in TGA results due to the surfactant degradation. Moreover, a mixture of these surface agents seems to shift the characteristic peak of ZnO to lower wavenumbers (blue-shifted), although calcinations treatment at 500 °C for 5 h could compensate for this effect to some extent. In addition, the cationic/anionic surfactant mixture modified the pore texture of ZnO especially for CTAB:SDS samples. Modifying the pore diameter and pore volume on one hand and decreasing the particle size on the other hand could enhance the surface area of the as-synthesized ZnO nanoparticles up to 29 m^2^/g.

## Supplementary Materials



## Figures and Tables

**Table 1:** TGA data of as-synthesized ZnO nanostructures synthesized at different mole ratios of (**A**) CTAB to SDS and (**B**) SDS to CTAB.

A. CTAB	Weight loss			Total W.L. %	Residue %
	onset	Offset	W.L. %		
0.0	34	502	7.8	7.8	92.6
0.5	65	443	3.2	3.5	96.6
	444 *	493 *	0.3 *		
1.0	70	478	4.7	4.7	94.3
1.5	70	650	5.2	5.2	95.0
2.0	38	215	1.7	7.0	92.6
	424 *	948 *	5.3 *		
**B. SDS**	**onset**	**Offset**	**W.L. %**	**Total W.L. %**	**Residue %**
0.0	35	456	5.3	5.3	93.5
0.2	31	455	5.6	5.6	94.8
0.36	247	934	2.2	2.2	99.0
0.5	30	349	4.8	8.0	92.2
	355 *	464 *	1.6 *		
	464 **	599 **	1.6 **		
1.0	105	152	2.6	12.5	86.4
	167 *	437 *	9.9 *		
1.5	79	592	5.6	5.6	94.6

*,** show second and third weight loss information, respectively.

W.L.: weight loss.

**Table 2:** BET surface area and BJH pore diameter and pore volume of as-synthesized ZnO nanostructures at different mole ratois of (**A**) CTAB to SDS and (**B**) SDS to CTAB.

CTAB	BET surface area (m^2^/g)	BJH pore diameter (nm)	BJH pore volume (cm^3^/g)
0.0	7	16.8	0.027
0.5	8	16.8	0.031
1.0	7	22.9	0.037
1.5	6	16.4	0.02
2.0	7	19.1	0.032

**SDS**	**BET surface area (m^2^/g)**	**BJH pore diameter (nm)**	**BJH pore volume (cm^3^/g)**

0.0	16	16.1	0.067
0.2	3	15.0	0.007
0.36	29	13.1	0.011
0.5	13	15.0	0.044
1.0	9	23.8	0.061
1.5	10	19.2	0.051

**Table 3:** BET surface area of the as-synthesized ZnO nanostructures synthesized at SDS:CTAB = 1:2 and CTAB:SDS = 1:0.36 at different temperatures, 120 °C, 150 °C and 180 °C.

Comparison in different temperature		

Sample	Temperature	Surface Area (m^2^/g)
ZnO(SDS:CTAB = 1:2)	120 °C	7
	150 °C	5
	180 °C	5

ZnO(CTAB:SDS = 1:0.36)	120 °C	29
	150 °C	2
	180 °C	2
