# Supplementary material for: The Effect of Sodium Dodecyl Sulfate (SDS) and Cetyltrimethylammonium Bromide (CTAB) on the Properties of ZnO Synthesized by Hydrothermal Method
Source: Int J Mol Sci. 2012 Oct 16;13(10):13275–93. doi: 10.3390/ijms131013275 (PMC3497326; doi:10.3390/ijms131013275)

Supplementary Information

Figure S1. TGA-DTG thermogravimetric analysis of as-synthesized ZnO samples prepared at different mole ratios of SDS:CTAB, (**a**) 1:0; (**b**)1:0.5; (**c**)1:1; (**d**)1:1.5; (**e**)1:2 and CTAB:SDS (**f**)1:0; (**g**)1:0.2; (**h**)1:0.36; (**i**)1:0.5; (**j**)1:1 and (**k**)1:1.5.

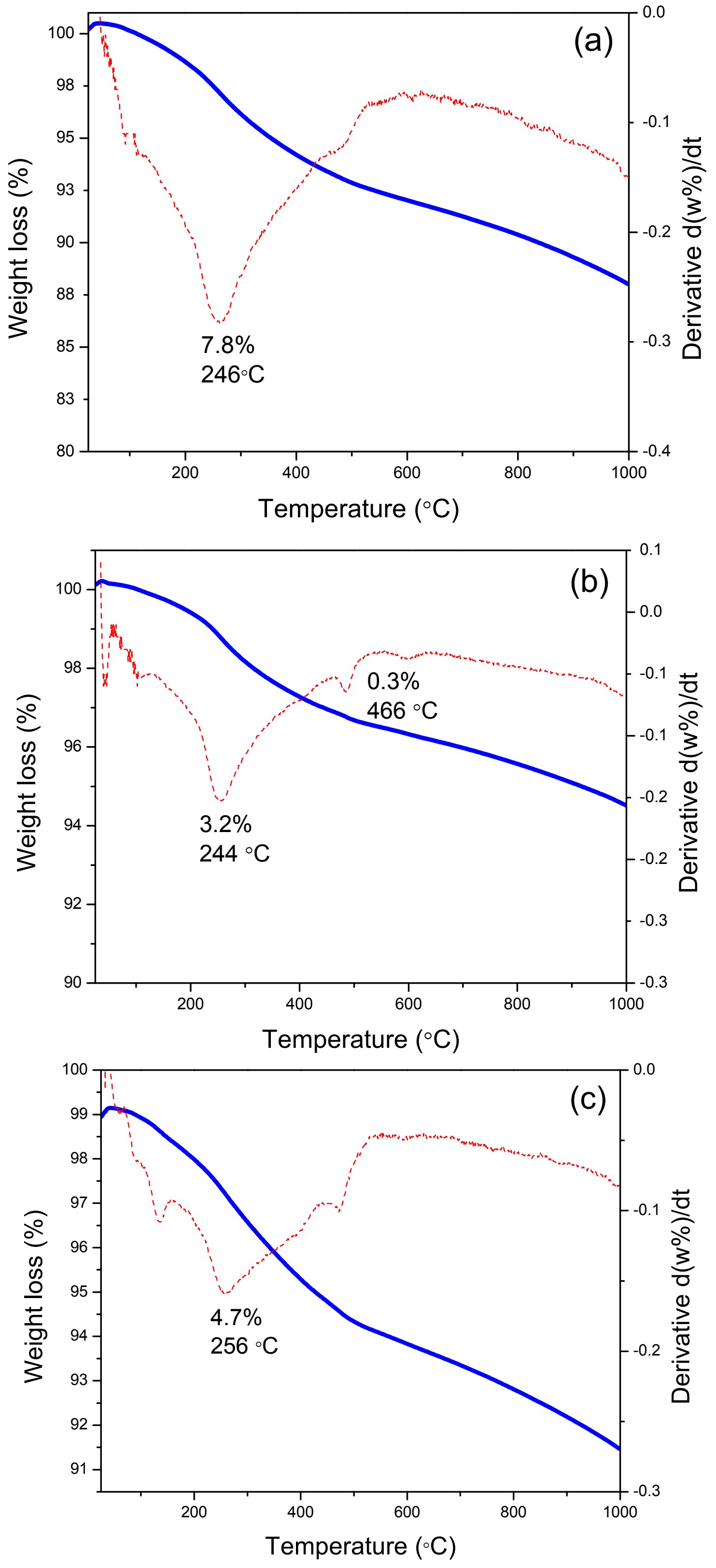

Figure S1. *Cont.*

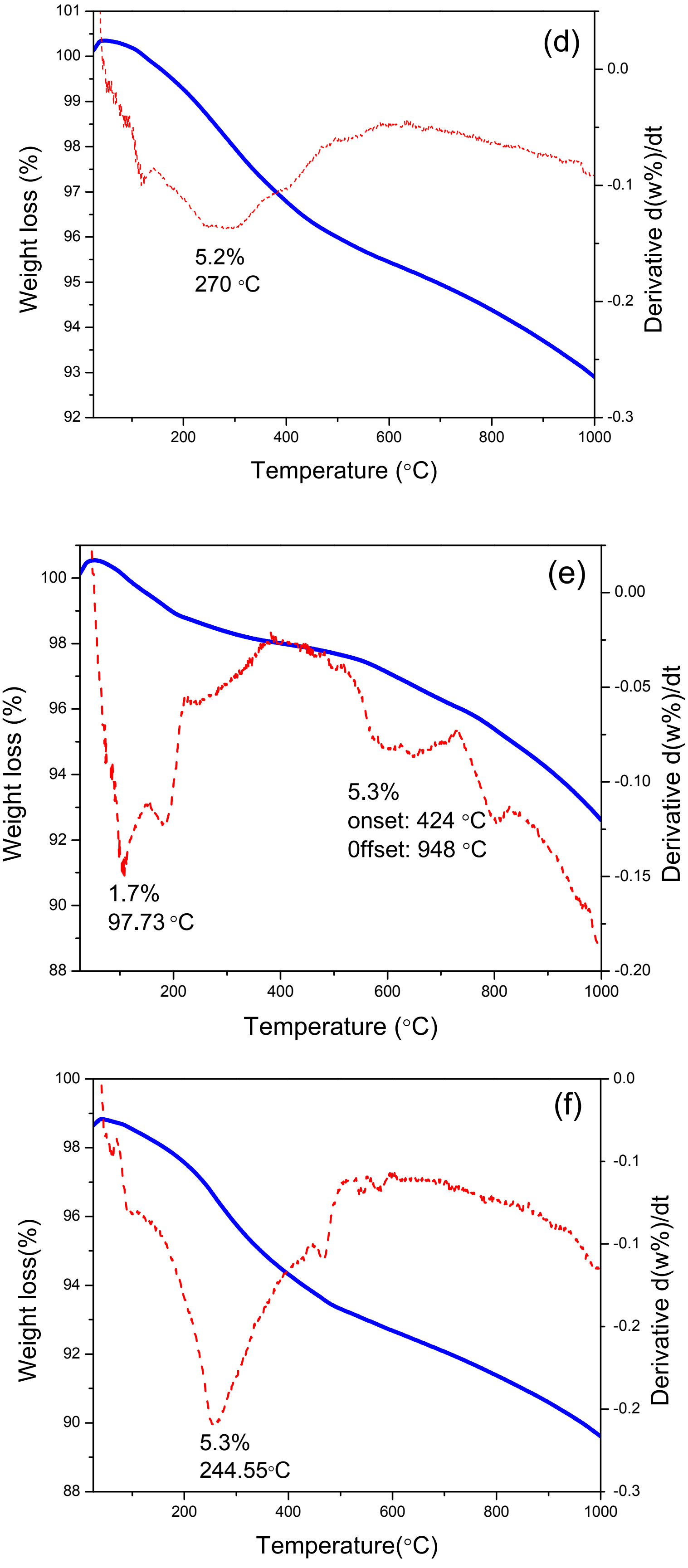

Figure S1. *Cont.*

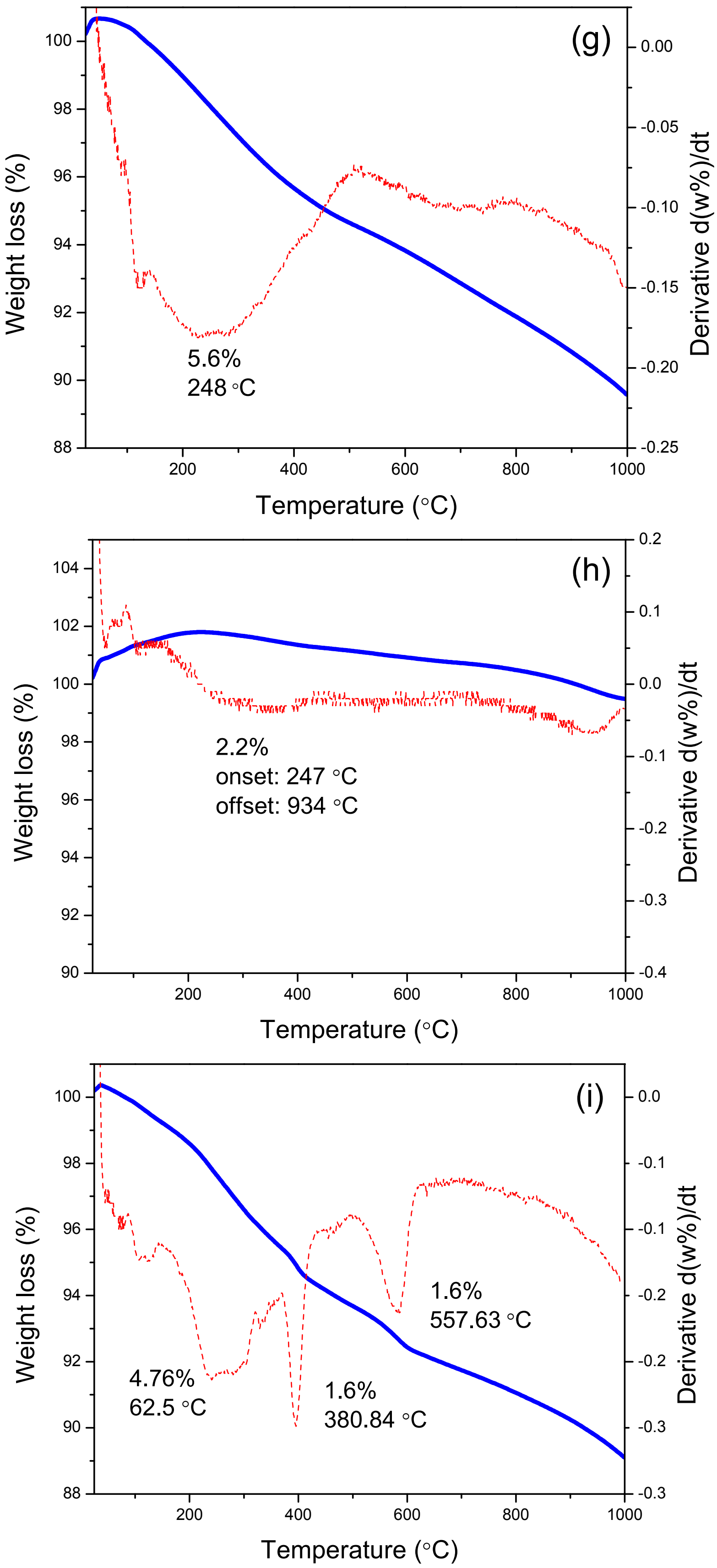

Figure S1. *Cont.*

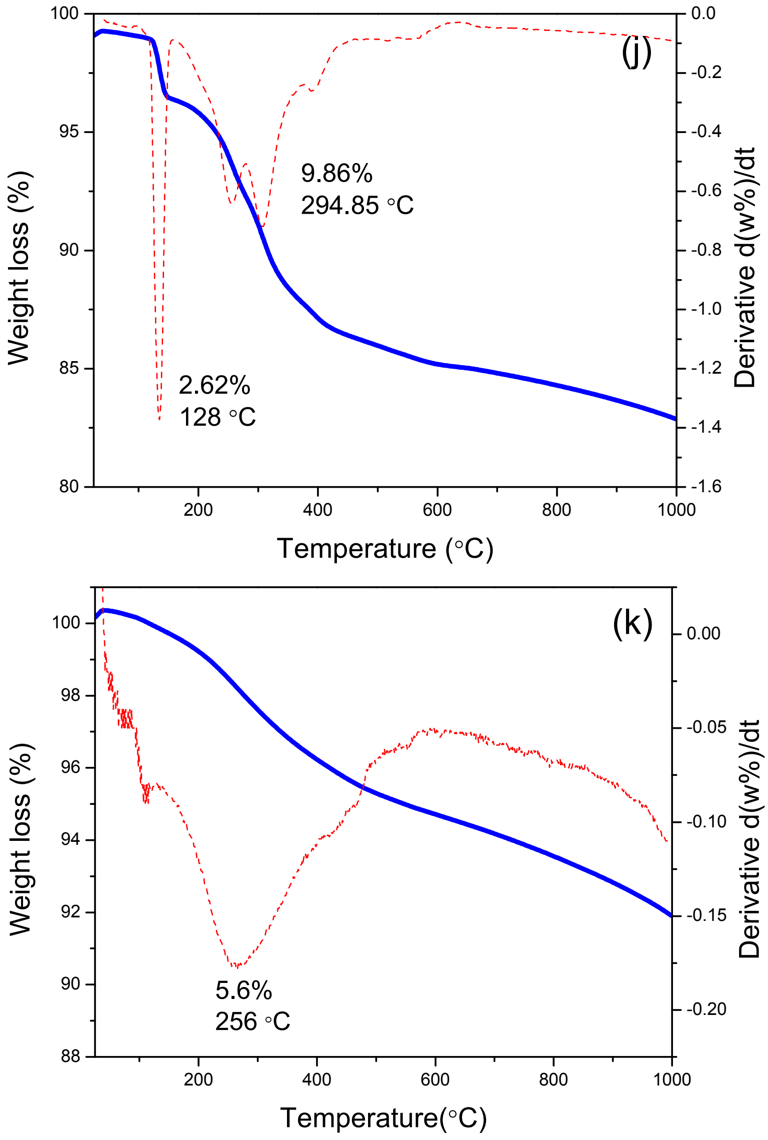

Supplement: Supplementary file 1 [file ijms-13-13275-s001.pdf]
